# Epithelial cells captured from ductal carcinoma *in situ* reveal a gene expression signature associated with progression to invasive breast cancer

**DOI:** 10.18632/oncotarget.12352

**Published:** 2016-09-30

**Authors:** Eliana Vanina Elias, Nadia Pereira de Castro, Paulo Henrique Baldan Pineda, Carolina Sens Abuázar, Cynthia Aparecida Bueno de Toledo Osorio, Mabel Gigliola Pinilla, Sabrina Daniela da Silva, Anamaria Aranha Camargo, Wilson Araujo Silva, Elisa Napolitano e Ferreira, Helena Paula Brentani, Dirce Maria Carraro

**Affiliations:** ^1^ Laboratory of Genomics and Molecular Biology, CIPE-International Research Center, A.C. Camargo Cancer Center, São Paulo, SP, Brazil; ^2^ National Institute of Science and Technology in Oncogenomics (INCITO), São Paulo, SP, Brazil; ^3^ Department of Anatomical Pathology, A.C. Camargo Cancer Center, São Paulo, SP, Brazil; ^4^ Ludwig Institute for Cancer Research, São Paulo, SP, Brazil; ^5^ Molecular Oncology Center, Sirio-Libanese Hospital, São Paulo, SP, Brazil; ^6^ Department of Genetics, Ribeirão Preto Medical School, University of São Paulo, Ribeirão Preto, São Paulo, SP, Brazil; ^7^ Institute of Psychiatry-Medical School, University of São Paulo (FMUSP), São Paulo, SP, Brazil

**Keywords:** breast cancer, ductal carcinoma *in situ* progression, gene signature, cellular-based analysis, molecular markers

## Abstract

Breast cancer biomarkers that can precisely predict the risk of progression of non-invasive ductal carcinoma *in situ* (DCIS) lesions to invasive disease are lacking. The identification of molecular alterations that occur during the invasion process is crucial for the discovery of drivers of transition to invasive disease and, consequently, biomarkers with clinical utility. In this study, we explored differences in gene expression in mammary epithelial cells before and after the morphological manifestation of invasion, i.e., early and late stages, respectively. In the early stage, epithelial cells were captured from both pre-invasive lesions with distinct malignant potential [pure DCIS as well as the *in situ* component that co-exists with invasive breast carcinoma lesions (DCIS-IBC)]; in the late stage, epithelial cells were captured from the two distinct morphological components of the same sample (*in situ* and invasive components). Candidate genes were identified using cDNA microarray and rapid subtractive hybridization (RaSH) cDNA libraries and validated by RT-qPCR assay using new samples from each group. These analyses revealed 26 genes, including 20 from the early and 6 from the late stage. The expression profile based on the 20 genes, marked by a preferential decrease in expression level towards invasive phenotype, discriminated the majority of DCIS samples. Thus, this study revealed a gene expression signature with the potential to predict DCIS progression and, consequently, provides opportunities to tailor treatments for DCIS patients.

## INTRODUCTION

Ductal carcinoma *in situ* (DCIS) is a type of non-invasive breast cancer in which tumor cells are confined within the ducts of the breast. DCIS is classically non-palpable, and many times this lesion is diagnosed incidentally during routine mammography. DCIS can be diagnosed as a pure DCIS lesion, which typically has an excellent prognosis, or it can be detected together with invasive breast cancer (DCIS-IBC). In DCIS-IBC, the prognosis is dictated by the invasive component, the true threatening lesion. The morphological aspects of the long-term natural history of DCIS require a multistep succession of histological changes including an initial premalignant stage of atypical ductal hyperplasia (ADH) that progress to the pre-invasive stage, i.e., DCIS, and culminates in invasive breast cancer (IBC), the potentially lethal stage [1]. The progression of DCIS to IBC has not yet been completely defined at the molecular level.

The traditional treatment for DCIS is surgery combined with radiation and hormonal therapy for patients with estrogen receptor*-*positive breast tumors; this treatment prevents progression to invasive disease and ensures high overall survival and disease-free survival rates [2]. The fraction of DCIS cases that progress to a threatening lesion, if left untreated, is imprecise and ranges from 14 to 50% [3, 4]. Intriguingly, most cases of DCIS progression are due to initial misdiagnosis as a benign lesion [4], suggesting that the true fraction of DCIS cases that would progress is substantially smaller. Recent data indicate that the disease-specific survival rate of patients with DCIS exceeds 98% [5], which, together with the characteristic indolence of this lesion, has forced the medical community to be aware of unnecessary overtreatment for most of DCIS patients. Unfortunately, the identification of patients with DCIS who are at high risk of progressing to an invasive cancer is not yet feasible, and consequently, the effectiveness of the available therapies for the treatment of each patient diagnosed with DCIS cannot be precisely determined [6]. Therefore, the current challenge is to discover molecular markers that can distinguish DCIS lesions with true potential to progress to invasive disease. Such markers would enable more adjusted and efficient therapy as well as the differentiation of patients who require greater clinical intervention to prevent tumor progression from patients with indolent tumors who would benefit from much more modest treatment.

Many groups have assessed the early molecular alterations that occur in epithelial cells within DCIS lesions during the transition of invasiveness [7–16], and a few genes that may be involved in this process have been identified [17–19]. Recent findings have indicated high similarities in gene expression profiles among epithelial cells captured from *in situ* and invasive regions of the same lesion [12, 20] and different lesions [21, 22]. This similarity has also been observed in mutational profiles [23] and in analysis of genomic abnormalities [24]. Additionally, greater changes have been observed in the gene expression of epithelial cells captured from two groups of *in situ* lesions (pure or co-existent with invasive lesion) than between cells captured from *in situ* and invasive lesions, which suggests that molecular alterations occur before the morphological manifestation of invasion [13].

Here, to widely assess the robust and subtle changes in gene expression that occur during progression from *in situ* to invasive breast cancer, we performed an epithelial cell-based gene expression analysis of the two stages of progression (before and after manifestation of invasion) using three-gene expression approaches: cDNA microarray, rapid subtractive hybridization (RaSH) library, and RT-qPCR array. We confirmed that the principal difference in gene expression is evident before the establishment of invasion, in the early stage, and identified a gene expression signature that discriminates the majority of samples according to their invasion abilities. Our data disclosed molecular mechanisms that underlie the two steps of DCIS progression and provide biomarkers with clinical potential for the prediction of the risk of invasion and personalized treatment of patients with DCIS.

## RESULTS

### Discovery of genes potentially involved in DCIS progression

**Figure 1:**
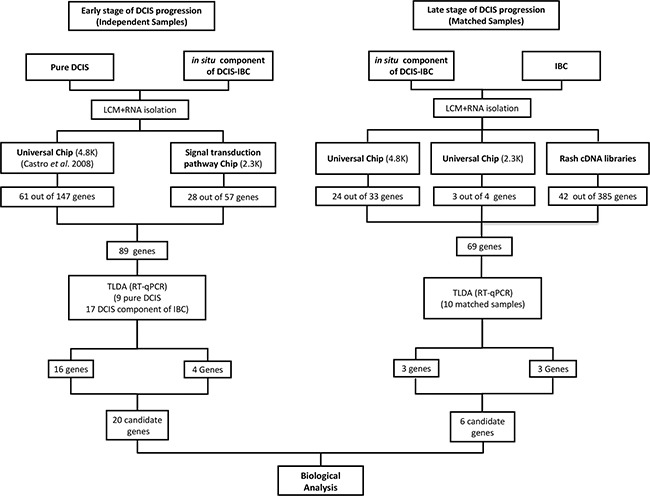
Workflow for candidate genes identification **(Left panel)**: Identification of differentially expressed genes between pure DCIS (without the presence of the invasive component) and the *in situ* component of DCIS-IBC samples. The 4.8K cDNA microarray platform was used to analyze 16 samples (5 pure DCIS and the *in situ* component of 11 DCIS-IBC samples) whereas the 2.3K cDNA microarray platform was applied for the analysis of 16 samples (4 pure DCIS and the *in situ* component of 12 DCIS-IBC). The criteria applied were fold-change ≥ |2| and *P-*value < 0.05. We selected 89 genes from these analyses for validation by RT-qPCR (TLDA assay) in 26 samples (9 pure DCIS and the *in situ* component of 17 DCIS-IBC samples). We confirmed that the expression of twenty genes was increased in pure DCIS (fold change ≥ |2| and *P*-value < 0.05). **(Right panel)**: Identification of differentially expressed genes between *in situ* and invasive components of DCIS-IBC matched samples. The 4.8K cDNA microarray platform was applied in the analysis of 16 DCIS-IBC matched-samples, whereas the 2.3K cDNA microarray platform was used for the 10 DCIS-IBC matched-samples. The criteria were fold change ≥ |1.5| and *P-*value < 0.05. The RaSH cDNA libraries were applied to analyze four matched-samples. We selected 69 genes from these analyses and subjected them to a validation by TLDA assay using 10 matched DCIS-IBC samples. We confirmed six differentially expressed genes (fold change ≥ |2| and *P*-value < 0.05). *Abbreviations:* DCIS, ductal carcinoma *in situ*; DCIS-IBC, *in situ* component of IBC; IBC, invasive breast carcinoma; RaSH, rapid subtractive hybridization; TLDA, TaqMan low-density arrays; LCM, laser capture microdissection.

We have evaluated molecular alterations in mammary epithelial cells in two distinct stages of DCIS progression: before (early stage) and after (late stage) the morphological manifestation of invasion. First, to discover genes potentially involved in the early stage of DCIS progression, we used a customized cDNA microarray platform (2.3K) enriched in genes belonging to signal transduction pathways [25]. We evaluated 16 samples (epithelial cells captured from 4 pure DCIS lesions and from the *in situ* component of 12 cases of DCIS-IBC) (Supplementary Table S1). In total, 57 genes were identified as differentially expressed between these two groups according to the criteria of fold change ≥|2| and *P-*value < 0.05. The majority (51 genes, 89%), exhibited increased expression in pure DCIS, suggesting predominant decrease in expression level toward DCIS progression. Of these genes, a set of 28 genes was randomly selected for validation by RT-qPCR array. We also incorporated in the validation array an additional set of 61 differentially expressed genes, obtained in our previous study [13] (Supplementary Table S2) in which we used a distinct customized cDNA platform (4.8K) and evaluated 15 samples (epithelial cells captured from 5 cases of pure DCIS and from the *in situ* component of 10 cases of DCIS-IBC) [13]. In total, we included 89 genes in the array (78 increased and 11 decreased in pure DCIS) (Figure 1, left panel; Supplementary Table S1) for further validation by RT-qPCR, keeping similar proportion of genes found in the discovery phase with decreased expression along the DCIS progression (88%).

Next, to discover genes potentially involved in the late stage of DCIS progression, we explored gene expression differences between epithelial cells from morphologically different components (*in situ* and invasive) of the same lesion. A set of 16 and 10 DCIS-IBC matched samples were analyzed by cDNA microarray using two customized platforms (4.8K and 2.3K platforms respectively) (Figure 1, right panel). Using the same criteria for genes involved in the early stage (fold change ≥|2| and *P-*value < 0.05), we identified only 8 differentially expressed genes on the 4.8K platform and none on the 2.3K platform (data not shown). Thus, to explore subtle differences in gene expression, we established less stringent criteria (fold change ≥ |1.5| and *P-*value < 0.05) and identified 33 differentially expressed genes on the 4.8K platform (28 with increased expression in the invasive component) and 4 genes on the 2.3K platform (3 with increased expression in the *in situ* component) (Supplementary Table S3; Figure 1, right panel). Of these genes, 27 were randomly selected for further validation by RT-qPCR array (24 of 33 genes from the 4.8K platform and 3 of 4 genes from the 2.3Kplatform).

In addition, we used RaSH cDNA libraries as an alternative methodology for the detection of subtle differences in gene expression during the late stage. RaSH cDNA library analysis is a sequence-based approach that is a sensitive method for the identification of rare transcripts with low expression levels [26]. In this method, transcripts common to two samples are excluded, resulting in a more robust process for the identification of genes with exclusive or increased expression in one of the samples (the tester cDNA library). Thus, we constructed subtracted cDNA libraries using mammary epithelial cells captured from the *in situ* and invasive components of 2 DCIS-IBC samples by adapting the RaSH method for amplified RNA (Supplementary Figure S1 - see Material and Methods for details). We identified 385 distinct transcripts that were detected exclusively in the epithelial cells (171 and 214 genes detected in *in situ* and invasive lesions, respectively). Of these transcripts, 42 were randomly selected (Supplementary Tables S4–S5), totalizing 69 genes selected for validation in the RT-qPCR array.

### Validation of candidate genes associated with the early and late stages of DCIS progression

To validate the set of 89 genes that were differentially expressed between distinct groups of pre-invasive lesions and are potentially involved in the early stage of DCIS progression (pure DCIS and the *in situ* component of DCIS-IBC), a custom Taqman low-density array (TLDA) was used (Supplementary Table S2). To increase the significance of our findings, we included microdissected samples from each group in the validation step: 5 new pure DCIS samples (total of 9 samples) and the *in situ* component of 4 new DCIS-IBC samples (total of 17 samples). Of the 89 genes, 20 (16 and 4 genes from the 4.8K and 2.3K platforms, respectively) were validated (fold change ≥ |2.0| and *P-*value < 0.05). Interestingly, all genes confirmed by TLDA exhibited decreased expression in the *in situ* component of the DCIS-IBC samples (Table 1, top section; Supplementary Figure S2).

**Table 1:** Differentially expressed genes in the early and late stages of DCIS progression confirmed by RT-qPCR approach

Samples	Gene ID	Gene name	pure DCIS	IC_DCIS-IBC	IBC	Fold change
**Early stage (pure DCIS/IC_DCIS-IBC)**	*ALMS1*	Alstrom syndrome 1	up	down	-	6.30
	*ANAPC13*	Anaphase promoting complex subunit 13	up	down	-	3.00
	*ARHGAP9*	Rho GTPase activating protein 9	up	down	-	10.20
	*AZGP1*	Alpha-2-glycoprotein 1, zinc-binding	up	down	-	4.30
	*CAMP*	cathelicidin Antimicrobial peptide	up	down	-	3.10
	*CHRNB1*	Cholinergic receptor, nicotinic, beta 1	up	down	-	3.10
	*CPNE3*	Copine III	up	down	-	2.80
	*CTTNBP2NL*	Cortactin-binding protein 2 N-terminal like	up	down	-	4.80
	*EDN1*	Endoglucanase1	up	down	-	5.76
	*EPOR*	Erythropoietin receptor	up	down	-	10.51
	*GRB10*	Growth factor receptor-bound protein 10	up	down	-	4.40
	*HLTF*	Helicase-like transcription factor	up	down	-	2.98
	*INPP1*	Inositol polyphosphate-1-phosphatase	up	down	-	2.62
	*LSM4*	U6 snRNA-associated Sm-like protein	up	down	-	3.53
	*MAPK8*	Mitogen-activated protein kinase 8	up	down	-	2.00
	*P4HB*	Prolyl 4-hydroxylase, beta polypeptide	up	down	-	21.32
	*RABEPK*	Rab9 effector protein with kelch motifs	up	down	-	2.17
	*RARRES3*	Etinoic acid receptor responder (tazarotene induced) 3.	up	down	-	3.38
	*REC8*	Meiotic recombination protein	up	down	-	9.50
	*UTP20*	Small subunit (SSU) processome component	up	down	-	2.79
**Late stage (DCIS-IBC)**	*CLNS1A*	Chloride channel, nucleotide-sensitive, 1A	-	down	up	2.77
	*FCGR3A*	Fc fragment of IgG, low affinity IIIa, receptor (CD16A)	-	down	up	3
	*POSTN*	Periostin, osteoblast specific factor	-	down	up	8.5
	*SAA1*	Serum amyloid A1	-	up	down	5.13
	*SLC37A1*	Solute carrier family 37 (glycerol-3-phosphate transporter), member 1	-	down	up	6.5
	*TFF1*	Trefoil factor 1	-	up	down	4.06

The top section shows differentially expressed genes between epithelial cells from pure DCIS and the *in situ* component of DCIS-IBC (early stage). A total of 20 genes exhibited a fold changes ≥ |2| and *P*-values < 0.05 by RT-qPCR (TLDA assay). The bottom section shows differentially expressed genes between epithelial cells from *in situ* and invasive components of matched DCIS-IBC samples (late stage). A total of 6 genes exhibited a fold changes ≥ |2| and *P*-values < 0.05 by RT-qPCR (TLDA assay). The increased and decreased expression (up and down) is based on the fold-change values obtained from the RT-qPCR validation. Abbreviations: DCIS, ductal carcinoma *in situ*; IC_DCIS-IBC, *in situ* component of DCIS-IBC; IBC, invasive breast carcinoma; Down, decreased expression; Up, increased expression; RT-qPCR, reverse transcription-quantitative polymerase chain reaction; TLDA, TaqMan low-density arrays

Subsequently, to validate the 69 differentially expressed genes identified in the late stage of DCIS progression (*in situ* and invasive components of DCIS-IBC matched samples), we used another custom TLDA (Supplementary Table S3). These genes were examined in 10 DCIS-IBC matched samples, including 4 novel microdissected samples. Of the 69 genes, only 6 were validated (3 genes from the RaSH assays and 3 genes from the 4.8K platform) (fold change ≥ |2|; *P-*value < 0.05) (Table 1, bottom section; Supplementary Figure S3).

### Identification of a gene expression signature of DCIS progression

**Figure 2:**
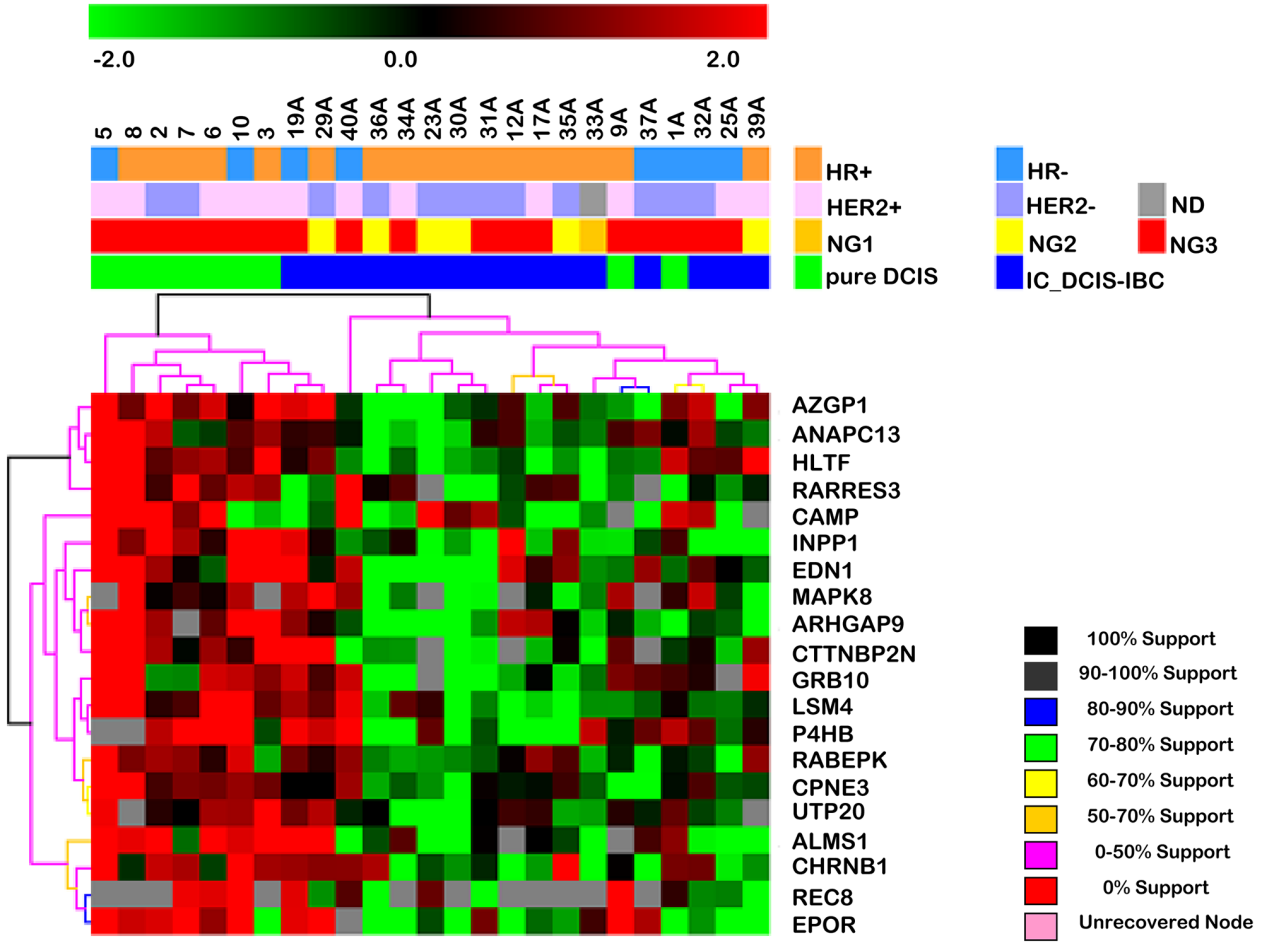
Hierarchical clustering based on the expression profile of the 20 differentially expressed genes in the early stage of DCIS progression (between pure DCIS and the *in situ* component of DCIS-IBC samples) Unsupervised hierarchical clustering with Euclidean distance and average linkage of 25 breast cancer samples, including 9 pure DCIS (green) and the *in situ* component of 16 DCIS-IBC samples (blue). The hierarchical clustering was based on log2-transformed expression values obtained from RT-qPCR validation assays. The columns and rows represent samples and genes, respectively. For each gene, the expression values were subtracted from the respective mean value of the row. Only genes with expression in at least 50% of the samples in each group were considered. Red and green indicate increased and decreased gene expression respectively. The genes listed exhibited expression changes 2-fold or greater with increased expression in pure DCIS. The dendrogram of the unsupervised hierarchical clustering of samples shows that this gene set allows the discrimination of the DCIS component of IBC from pure DCIS samples. Abbreviations: DCIS, ductal carcinoma *in situ*; IC_DCIS-IBC, *in situ* component of DCIS-IBC; HER2, human epidermal receptor 2; HR, hormonal receptor (estrogen and progesterone receptors); ND, FISH non-determined; NG, nuclear grade.

To identify a gene expression signature associated with DCIS progression, we used unsupervised hierarchical clustering to evaluate the ability of the expression profile of each set of genes to correctly discriminate the samples. The expression profile of the early-stage genes distinguished 78% of the epithelial cell captured from pure DCIS samples and 89% of cells captured from the *in situ* component of DCIS-IBC samples (Figure 2). Furthermore, we verified whether this gene expression signature was able to correctly discriminate samples exclusively from the validation group (9 novel samples: 5 pure DCIS and 4 *in situ* component of DCIS-IBC samples) and obtained similar results; 80% of pure DCIS samples were correctly distinguished from 100% of the *in situ* component of DCIS-IBC samples (Supplementary Figure S4). However, given the small number of novel samples used in the validation phase, due to the low availability of pure DCIS frozen samples, this gene signature must be assessed further using in a wider sample set in order to define its precise prognostic value. In contrast, as expected, the gene expression profile representative of the late stage did not accurately discriminate between samples even when we evaluated cells from non-matched samples (data not shown).

### Assessment of the regulatory interconnection of the two gene sets

To understand the regulatory interconnection among the 26 genes potentially involved in the two steps of DCIS progression, we used IPA software. This analysis generated two significant gene networks associated with cell signaling-related functions. Network 1 (Supplementary Figure S5A) was overrepresented by genes involved in cell death and survival, cell signaling, cellular function and maintenance and exhibited an interconnection among 13 of the 26 genes, including 9 from the early stage (*AZGP1*, *CAMP*, *EDN1*, *EPOR*, *GRB10*, *INPP1*, *MAPK8*, *P4HB* and *RARRES3* with decreased expression toward invasion capability) and 4 from the late stage (*POSTN* with increased expression toward invasion capability - and *FCGR3A, SAA1* and *TFF1* with decreased expression toward invasion capability). These genes were highly interconnected, and *EDN1* and *MAPK8* were in the center of the network, which, in turn, are associated with mitogen-activated protein kinases (MAPKs) which may participate in the signaling cascades that regulate cellular processes such as cell proliferation, differentiation, and apoptosis. Network 2 (Supplementary Figure S5B) was overrepresented by genes involved in the cell cycle, reproductive system development and function as well as cellular development and exhibited an interconnection among 13 of the 26 genes, including 11 from the early stage (*ALSM1, ANAPC13, ARHGAP9, CHRNB1, CPN3, CTTNBP2NL, HLTF, LSM4, RABEPK, REC8,* and *UTP20* with decreased expression toward invasion capability) and 2 from the late stage (*CLNS1A* and SLC37A1 with increased expression toward invasion capability). These genes were sparsely interconnected and were related to UBC (ubiquitin C), a gene associated with protein degradation, DNA repair, and cell cycle regulation [27]

## DISCUSSION

A major goal for the treatment of DCIS is the prevention of the development of invasive disease. No biomarkers with clinical value are available that can accurately identify cases of DCIS at high risk for progression to invasive breast cancer. Thus, a meticulous investigation of the molecular alterations that underlie the invasion process may benefit the identification of potential biomarkers to successfully tailor the clinical management of women with DCIS. Therefore, we performed a comprehensive epithelial cell-based analysis of two stages of DCIS progression, before and after the morphological manifestation of invasion to identify potential driver genes involved in DCIS progression.

Extensive study of the transition of DCIS to invasive ductal carcinoma has revealed slight molecular differences in the epithelial cells of these morphologically distinct lesions. These findings suggested that the ability of epithelial tumor cells confined to the duct of breast tissue to invade the surrounding tissue is driven by either slight changes in gene expression that cannot be detected using conventional approaches, or that occur before the morphological appearance of invasion [13]. To examine the first possibility, we used the RaSH approach [26], a much more sensitive tool that is appropriate for the identification of previously undetected expression differences that might occur in epithelial cells in the late stage of DCIS progression (*in situ* to invasive transition). Although a few genes were detected using this sensitive methodology, in agreement with other studies [12, 20, 28], the expression patterns of these genes did not correctly discriminated between samples. Thus, our current results support the notion that these two histologically distinct lesions are highly similar at both genetic and molecular levels. To assess the second possibility, we expanded our initial analysis [13] using an additional microarray platform with genes enriched for signal transduction pathways [25] and included new samples in the validation phase, observing greater differences in gene expression modulation, in accordance with our previous study [13]. Moreover, this set of 20 genes of the early stage defined a signature that discriminated the majority of epithelial tumor cells from the two distinct groups of pre-invasive lesions, consistent with the hypothesis that molecular alterations are established prior to the morphological manifestation of invasion. Interestingly, in agreement with our previous findings [13], we also detected a preferential decrease in gene expression in the early stage of DCIS progression, which suggests that the invasion capability that is manifested in epithelial tumor cells in the early phase of the process may be enhanced by the inactivation of tumor suppressor genes.

The tumor microenvironment, which comprises diverse cell types, likely plays a fundamental role in tumor progression [16, 29] and with respect to breast cancer, the roles of myoepithelial and fibroblast cells in mammary tumor progression have been increasingly recognized [14]. Our results are limited to the assessment of luminal epithelial cells, and the role of tumor microenvironment cells in the two steps of DCIS progression, before and after the invasion manifestation, remains to be addressed.

Functional analysis of the regulatory interconnection among the genes modulated in both steps of DCIS progression revealed that 50% of the genes (13 out of 26) were related to cell-to-cell signaling and interaction, and the resulting network included highly interconnected genes such as *EDN1* and *MAPK8*. Both genes are associated with players in important cancer-related pathways, including *p38 MAPK* [30], *PI3K* [31], *ERK1/2* [32], *JNK* [32] and *TGF-beta* [33]. *EDN1* and *MAPK8* are also critical genes for TGF-beta-dependent induction of EMT [34]. *MAPK8* is primarily activated by cytokines, which can phosphorylate paxillin, a focal adhesion adaptor required for the formation of focal adhesion plaques and cell migration [35]. Increased expression of *MAPK8* upon loss of its direct transcriptional repressor *KLF4* induces apoptosis [36]. EDN-1 is an endothelial cell-derived vasoconstrictor peptide that has been associated with the development of several cancers through the activation of pathways involved in cell proliferation, migration, invasion, EMT, osteogenesis and angiogenesis [37]. Additionally, circulating levels of EDN-1 precursor have been suggested as a potential biomarker for the early diagnosis of breast cancer [38].

Other early-stage candidate genes in this network that may be involved in tumorigenesis, include *RARRES3*, *GRB10*, *INNP1* and *EPOR*. *RARRES3* is thought to act as a tumor suppressor or growth regulator that suppresses metastasis; it has also been associated with the modulation of the acylation status of Wnt proteins to suppress EMT and cancer stem cell properties [39]. Similarly, recent studies have determined that GRB10 interferes with the binding of Wnt proteins on the cell surface, indicating a possible molecular mechanism by which overexpression of GRB10 suppresses Wnt signaling [40]. Moreover, increased expression of GRB10 inhibits the PI3K/AKT and MAPK pathways, whereas GRB10 deficiency increases the insulin-dependent phosphorylation of proteins within these pathways, including AKT and MAPK1 [41, 42]. On the other hand, the current knowledge of *INPP1* and *EPO* is inconsistent with their expression behavior in our study. Evidences show that inactivation of INPP1 leads to a reduction in glycolytic intermediates; this reduction impairs cancer cell motility, invasiveness, and tumorigenicity via a complex feed-forward mechanism [43]. Similarly, high expression of EPO and its receptor, EPOR, has recently been described in breast cancer cells [44, 45], and in regions of hypoxia in breast tumors, suggesting that this gene may be involved in breast tumorigenesis by promoting tissue hypoxia [44]. Tumor hypoxia has been frequently associated with tumor propagation and tumor cell dissemination. Thus, investigation in depth is compulsory to precisely define the function of these genes in the early stage of DCIS progression.

Among the studies that investigated the molecular basis of DCIS progression [10, 12, 13, 19], little overlap has been detected with our signature. Exceptions are *POSTN*, and *ANAPC13* genes*. POSTN* (*Periostin*), a mesenchyme-specific gene, has been recurrently identify as highly expressed in invasive breast cancer, in agreement with our results [9, 12, 19]. POSTN promotes tumor progression and angiogenesis mediated by the VEGF receptor Flk-1/KDR [46]. POSTN also promotes survival in colon cancer via the activation of AKT and to the integrin α_v_β_3_-focal adhesion kinase (FAK)-mediated signaling pathway [47]. Malanchi *et al.* [48] demonstrated that, due to its ability to interact with Wnt ligands and activate the Wnt pathway, POSTN acts in metastatic colonization by modulating the interactions between breast cancer stem cells and their metastatic niche. More recently, polymorphisms in the *POSTN* gene have been associated with breast cancer susceptibility [49]. Further support was provided by our IPA analysis, which revealed interconnections of this gene with collagen and matrix metalloproteinases, reinforcing the hypothesis that this gene is associated with cancer cell invasiveness and metastasis. ANAPC13 encodes a component of the anaphase-promoting complex (APC/C) [50] which controls cell cycle progression. Decreased expression of ANAPC13, at protein and transcript level in tumor, was associated with poor survival in patients with invasive breast tumor and with higher genomic instability in invasive breast tumors, respectively [15].

Altogether, the IPA analysis provided an initial functional perspective of the mechanism that may underlie the transition of *in situ* breast cancer to invasive breast cancer. However, the true effect of the variations of the expression of these genes, both individually and in conjunction with the context of DCIS progression, requires additional investigation.

In summary, using comprehensive gene expression analysis, we have demonstrated that substantial changes in the expression patterns of genes involved in DCIS progression occur in epithelial tumor cells in the early stage of the process, before the morphological manifestation of invasion. We have also identified a gene signature with a predominant decrease in expression level toward the invasion capability, which suggests that the invasion process may be marked by the inactivation of tumor suppressor genes. The true roles of the genes uncovered in this study remain to be determined. Additionally, the clinical utility of this gene set for the discrimination of DCIS lesions at risk for progression to invasive disease and, consequently, for the personalization of treatment requires additional analyses using a larger cohort of DCIS patients with long-term follow-up.

## MATERIALS AND METHODS

### Gene identification strategy

We evaluated the molecular alterations that occur in epithelial cells in the two distinct stages of DCIS progression: before (early stage) and after (late stage) the morphological manifestation of invasion. The early stage refers to the molecular alterations between the two types of pre-invasive lesions with distinct malignant potential (cells captured from pure DCIS and from the *in situ* component of DCIS-IBC). The late stage refers to the molecular alterations in epithelial cells captured from the two morphological distinct components, *in situ* and invasive, of the same tumor (matched DCIS-IBC samples). The matched sample design was used in order to compare epithelial cells with the same genetic background and thus increase our chances of identifying differences in gene expression that are involved in the morphological manifestation of invasion. Epithelial cells were captured by laser microdissection, and their transcriptional profiles were assessed in a comprehensive manner using two different approaches - cDNA microarray and rapid subtractive hybridization (RaSH) cDNA libraries - to discover potential differentially expressed genes which were validated by RT_qPCR as described in the flowchart in Figure 1 (Left panel, selection of putative genes involved in the early stage of DCIS progression; right panel, selection of putative genes involved in the late stage of DCIS progression).

### Tumor samples – cases, laser capture microdissection and total RNA purification

Frozen samples from 42 cases (63 breast specimens) were subjected to laser capture microdissection (LCM) (Supplementary Table S1). The inclusion criterion was female patients with ductal carcinoma without preoperative systemic treatment. Samples were obtained from the Biobank of A.C. Camargo Cancer Center, São Paulo, Brazil. For the pure DCIS samples, all hematoxylin and eosin (H&E)-stained slides for each patient were examined by a pathologist (CABTO) to ensure the absence of any previously undetected microinvasion. DCIS samples were classified according to the World Health Organization guidelines. For IBC samples, the Nottingham (Elston-Ellis) modification of the Scarff-Bloom-Richardson grade system (SBR grade) was applied. All breast cancer samples were previously analyzed by immunohistochemistry (IHC) for the expression of estrogen receptor (ER) (rabbit monoclonal anti-ER, clone SP1; Dako, Carpinteria, CA, USA), progesterone receptor (PR) (mouse monoclonal anti-PR, clone PgR636; Dako) and human epidermal growth factor receptor type 2 (HER2) (rabbit polyclonal anti-HER2, 1:1000; Dako), as routinely performed at our Cancer Center. ER, PR, and HER2 status was established according to the recommendations of the American Society of Clinical Oncology and the guidelines of the College of American Pathologists [51, 52]. Fluorescent *in situ* hybridization (FISH) analysis was performed in HER2 positive 2+ IBC samples according to the manufacturer's instructions (Dako).

Approximately 3,000–5,000 epithelial cells from pure DCIS lesions and from both the *in situ* and invasive components of DCIS-IBCs were captured by laser microdissection using the PixCell II LCM system (Arcturus Engineering, Mountain View, CA, USA) [53]. Frozen sections with a thickness of 4–7 μm were mounted onto glass slides and stained with the Arcturus® HistoGene® LCM Frozen Section Staining Kit (Arcturus Engineering); cells were captured using CapSure® HS LCM Caps (Arcturus Engineering) according to the manufacturer's protocol. Total RNA from epithelial cells was extracted using the PicoPure ™ RNA Isolation kit (Arcturus Engineering #KT0204), which included a DNase treatment step using the RNase-Free DNase Set (Qiagen, Germantown MD USA) according to the manufacturer's recommendations. This study was approved by the Ethics Committee of the Medical and Research Center of A.C. Camargo Cancer Center/SP under protocol numbers 587/04 and 708/05.

### Rapid Subtractive Hybridization (RaSH) cDNA libraries

The RaSH protocol was based on work by Jiang and colleagues [26] with several modifications for the use of amplified RNA (aRNA) due to the insufficient amount of RNA (Supplementary Figure S1) extracted from the captured cells. In summary, for first-strand cDNA synthesis, total RNA was mixed with 200 ng of dT-T7-GATC oligonucleotide [an oligonucleotide containing the GATC sequence (*Dpn* II endonuclease recognition site), 5' GGC CGA TGA ATT GTA ATA CGA CTC ACT ATA GGG AGG CGG **GAT C**TT TTT TTT TTT TTT TTT TTT TTT T 3'], 1X first-strand buffer (Invitrogen Life Technologies, Carlsbad, CA, USA), 200 mM DTT (Invitrogen Life Technologies), 20 mM dNTP (Invitrogen Life Technologies), 40 U RNasin® ribonuclease inhibitor (Promega, Madison, WI, USA) and 400 U of SuperScript II reverse transcriptase (Invitrogen Life Technologies). For double-strand cDNA (ds-cDNA), the second-strand cDNA synthesis was performed by adding 200 ng of template-switch-GATC oligonucleotide (TS-GATC) (an oligonucleotide containing the GATC sequence, 5' AAG CAG TGG TAA CAA CGC AGA **GAT C**GC GGG 3'), 1X Advantage PCR buffer, 20 mM dNTP, 2 U of RNase H, and 5 U of Advantage polymerase in a final volume of 100 μL. cDNA purification and precipitation were performed using the phenol-chloroform and ethanol methods, respectively. *In vitro* transcription was performed in the presence of purified ds-cDNA, 1X reaction buffer, 7.5 mM rNTP and 2.5 μL of T7 enzyme mix for 6 h at 37°C to generate antisense amplified RNA (aaRNA). The aaRNA was purified using TRIzol reagent (Invitrogen Life Technologies) according to the manufacturer's recommendations. Next, to convert aaRNA to ds-cDNA, the aaRNA was mixed with both 9 μg of random hexamer oligonucleotide (dN6) and 300 ng of TS-GATC oligonucleotide for the first strand and with 300 ng of dT-T7-GATC oligonucleotide for the second strand, using the same conditions described previously. The subsequent steps for the generation of the subtracted cDNA libraries were performed following the original RaSH protocol using a proportion of 50:1 (driver:tester cDNA populations) [26]. The tester cDNA population (*Xho* I-digested) is a cDNA population whose common transcripts were subtracted from the cDNA driver population (*Xho* I-non digested). The resulting subtracted cDNA population was cloned to generate a cDNA library enriched for transcripts that are increased in the cDNA tester population. Two matched DCIS-IBC samples were used to generate the four subtracted cDNA libraries (two DCIS_tester and two IBC_tester libraries). DNA sequencing was performed using the BigDye Terminator v3.1 Cycle Sequencing Kit (Applied Biosystems, Carlsbad, CA, USA) in an ABI 3100 sequencer. Sequence reads were analyzed using a customized pipeline for vector trimming and masking of repetitive elements using the RepeatMasker program. In total, 1,099 reads that matched the RefSeq, Unigene or EST databases were selected. Finally, a manual inspection was performed, and 385 transcripts that were identified in only one of the two components of DCIS-IBC were selected (Supplementary Tables S4–S5). Reads exclusively identified in one of the testers were assumed to be activated in the corresponding DCIS-IBC matched component.

### cDNA microarray experiments

The two-round linear amplification procedure was based on T7-driven amplification as previously described [13]. Total RNA from HB4a normal human mammary luminal epithelial cells [54] was extracted and amplified according to the same protocol and used as a reference for microarray competitive co-hybridizations. The cDNA labeling, hybridization, data normalization and analysis were performed as previously described [13].

Two customized cDNA microarray platforms were used in this study. The first was a universal chip (4.8K platform), comprising 4,608 human genes [described on the Gene Expression Omnibus website (http://www.ncbi.nlm.nih.gov/geo) under accession number GPL1930] [13, 55, 56]. The second was a signal transduction pathway chip (2.3K platform), comprising 1,512 human genes of distinct pathways including the epithelial-mesenchymal transition (EMT.) WNT signaling and the phosphatidylinositol-3-kinase (PI3K) pathways as well as additional human genes, as previously described [57].

### RT quantitative PCR experiments (RT-qPCR)

To validate the differentially expressed genes, complementary DNA was synthesized from 1 μg of amplified RNA [58], as described previously [13], because amplified RNA does not result in any bias in relative expression data [59]. Reverse-transcription reactions were performed using random dN6 primers or oligo (dT) and Superscript III enzyme (Invitrogen Life Technologies) following the manufacturer's recommendations. Gene quantification was performed by Custom TaqMan Low-Density Array (TLDA) experiments (Applied Biosystems) using pre-designed assays selected according to the following criteria: 3' end human inventoried assays encompassing two different exons and complementary to many variants. We designed 2 TLDA assays, one for early-stage gene candidates and a second for late-stage candidates. Each assay was performed using cDNA synthesized from two-round amplified RNA as described previously [13] and 1X TaqMan Universal PCR Master Mix according to the manufacturer's instructions (Applied Biosystems).

### Mathematical and statistical analyses

For microarray analysis, pairwise Student's t-tests were conducted to identify differentially expressed genes between distinct pathological lesions of matched DCIS-IBC samples (*in situ* and invasive components of DCIS-IBC), whereas unpaired Student's t-tests were conducted for independent samples (pure DCIS and the *in situ* component of DCIS-IBC). Differentially expressed genes were defined based on fold changes ≥ |2| and *P*-values < 0.05. Unsupervised hierarchical clustering analysis using MeV (Multiple Experiment Viewer) 4.4.1 software was applied using Euclidean distance and average linkage. The reliability of clustering was assessed using the bootstrap technique. For this analysis, we used genes expressed in at least 50% of the samples in each analyzed group.

For RT-qPCR analysis, relative quantitation was performed using an Applied Biosystems 7900HT System. We evaluated the expression of 6 control genes for the early stage (*ACTB*, *BCR*, *GAPDH*, *GUSB*, *HPRT1*, and *RPLPO*) and 5 control genes for the late stage (*B2M*, *GUSB*, *HPRT1*, *RPLPO* and *18S*) in TLDA assays and used geNorm v3.5 [60] to identify the most suitable control genes based on M values < 1.5. Data were normalized by calculating the ratio between the 2^−Cq^ of the target gene and the 2^−Cq^ of the control gene(s) [61]. The fold change was obtained by comparing the normalized mean values between the sample groups (pure DCIS, the *in situ* component of DCIS-IBC and IBC). RT-qPCR data analysis was performed using the GraphPad Prism program (Version 5.0, GraphPad Software) and normalized expression values were converted to a logarithmic scale using a log base 2. The statistical significance of relative gene expression between data sets was analyzed by an unpaired Student's t-test for independent samples and a paired Student's t-test for matched samples. Differentially expressed genes were defined based on fold changes ≥ |2| and *P*-values<0.05.

To perform functional enrichment analysis of the differentially expressed genes potentially involved in DCIS progression, we applied the core analysis of the Ingenuity Pathway Analysis (IPA) system (Qiagen), which provides a comprehensive resource based on manual collection and curation. Default parameters were used to identify enriched networks as well direct and indirect regulatory interactions that were predicted with high confidence and/or that were previously experimentally verified. The significance was determined by a default threshold [(*P*-value < 0.05) (−log [*P*-value]>1.3) (without application of the Benjamini-Hochberg multiple testing correction)].

## SUPPLEMENTARY FIGURES AND TABLES










