# Supplementary material for: Epithelial cells captured from ductal carcinoma in situ reveal a gene expression signature associated with progression to invasive breast cancer
Source: Oncotarget. 2016 Sep 30;7(46):75672–84. doi: 10.18632/oncotarget.12352 (PMC5342769; doi:10.18632/oncotarget.12352)
Supplement: Supplementary file 1 [file oncotarget-07-75672-s001.pdf]

Epithelial cells captured from ductal carcinoma *in situ* reveal a gene expression signature associated with progression to invasive breast cancer

SUPPLEMENTARY FIGURES AND TABLES

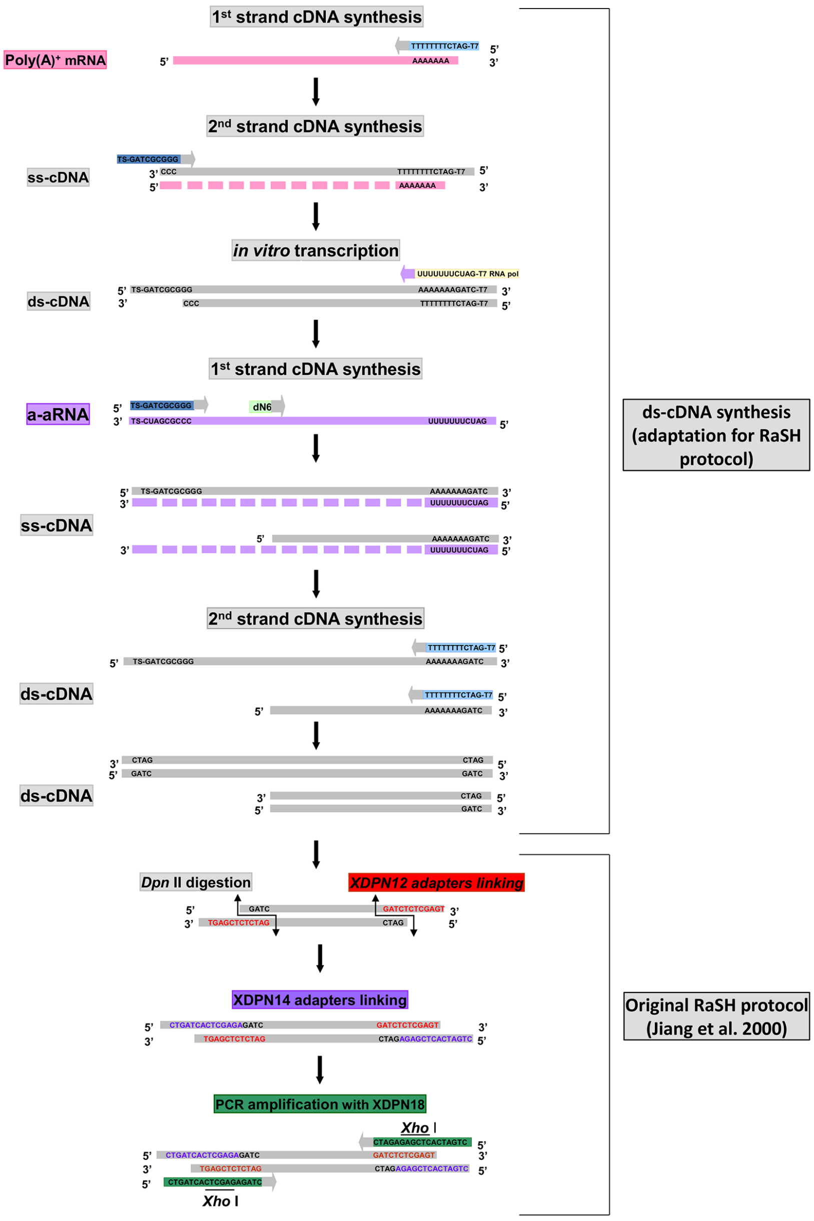

Supplementary Figure S1: Gene identification strategy adopted in RaSH cDNA libraries. The strategy adopted to identify differentially expressed genes in matched DCIS-IBC samples according to RaSH approach, with few modifications for using amplified RNA, which includes the LCM of epithelial cells and total RNA isolation. The cDNA synthesis was adapted for following *in vitro* transcription and another cDNA synthesis reaction by applying oligonucleotides with GATC target sequences. Next, it was performed a *Dpn* II digestion, adapters linking and PCR amplification. The subtractive hybridization consisted in 50 times more driver than tester cDNA population. The tester cDNA population (the differentially expressed genes) was cloned into previously *Xho* I digested pZERO plasmid. The constructs were screened and sequenced and sequences analyzed by a customized pipeline. Abbreviations: DCIS, ductal carcinoma *in situ*; IBC, invasive breast carcinoma; LCM, laser capture microdissection; RaSH, rapid substractive hybridization.

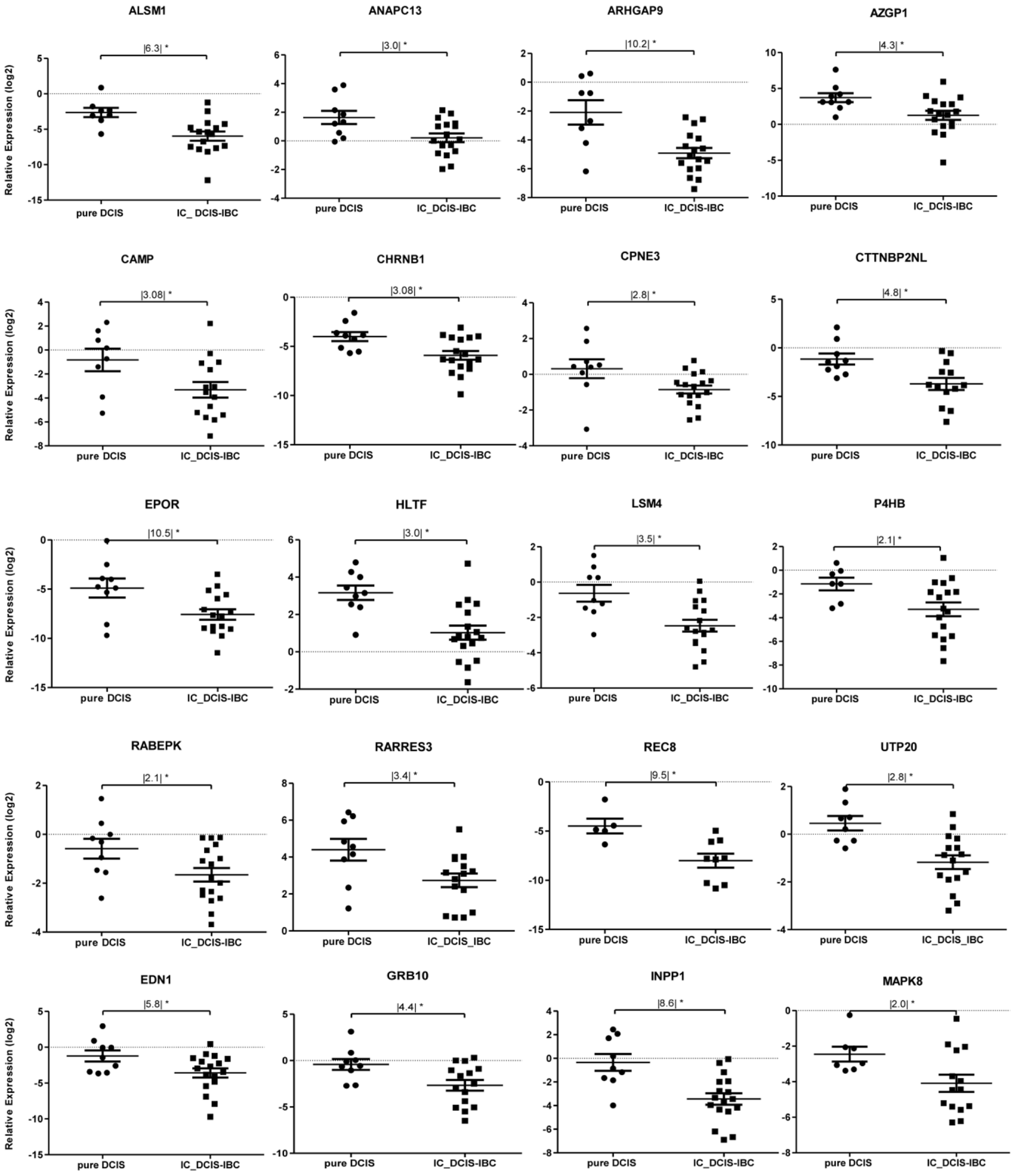

Supplementary Figure S2: Box plots for the 20-gene signature showing gene expression changes between pure DCIS and *in situ* component of DCIS-IBC samples obtained by RT-qPCR experiments. Data analysis was performed with GraphPad Prism program (Version 5.0, GraphPad Software), using normalized expression values converted to a logarithmic scale using a log base 2 (Y-axis). Sample groups are given on the X-axis. Statistical significance of relative gene expression between data sets was analyzed applying an unpaired Student's t-test for independent samples. Differentially expressed genes were defined following the criteria of fold change ≥ |2| and *P*-value<0.05. Fold change values between pure DCIS and *in situ* component of DCIS-IBC samples are indicated with absolute value bars | |. Asterisk (*) indicate *P*-value<0.05. Abbreviations: DCIS, ductal carcinoma *in situ*; IC_DCIS-IBC, *in situ* component of DCIS-IBC; IBC, invasive breast carcinoma.

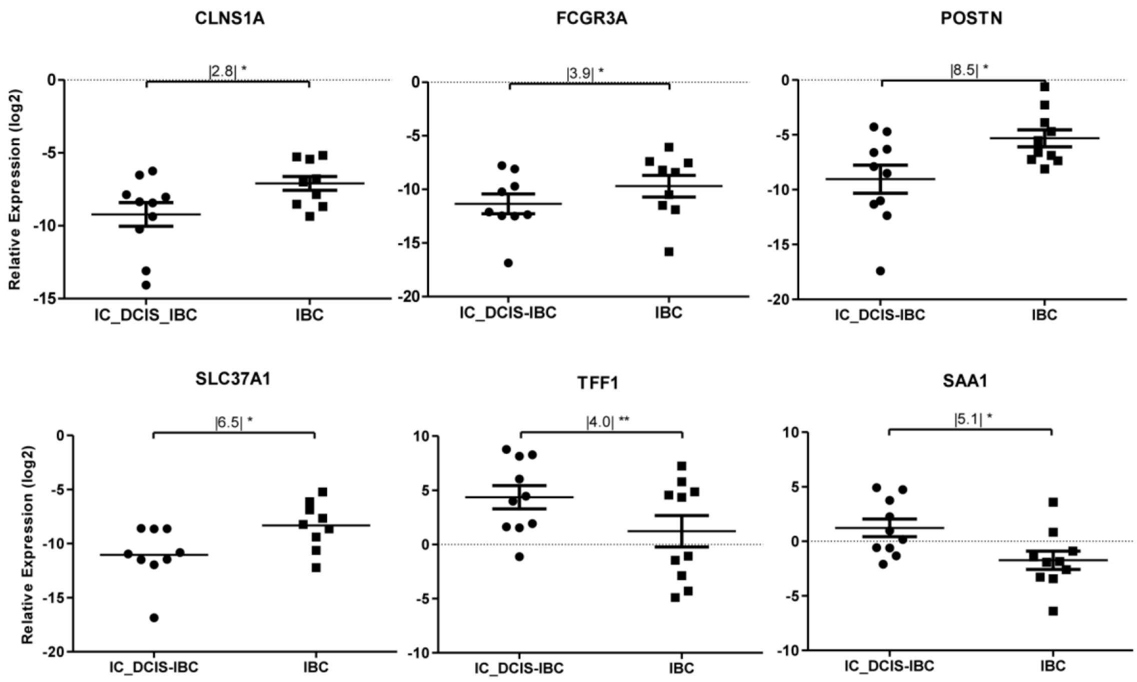

Supplementary Figure S3: Box plots for the 6-gene signature showing gene expression changes in matched DCIS-IBC samples obtained by TLDA assay. Data analysis was performed with GraphPad Prism program (Version 5.0, GraphPad Software), using normalized expression values converted to a logarithmic scale using a log base 2 (Y-axis). Sample groups are given on the X-axis. Statistical significance of relative gene expression between data sets was analyzed applying an unpaired Student's t-Test for independent samples. Differentially expressed genes were defined following the criteria of fold change ≥ |2| and P-value<0.05. Fold change values between DCIS and IBC samples are indicated with absolute value bars | |. Asterisk (*) indicate P-value<0.05. Abbreviations: DCIS, ductal carcinoma *in situ*; IC_DCIS-IBC, *in situ* component of DCIS-IBC; IBC, invasive breast carcinoma.

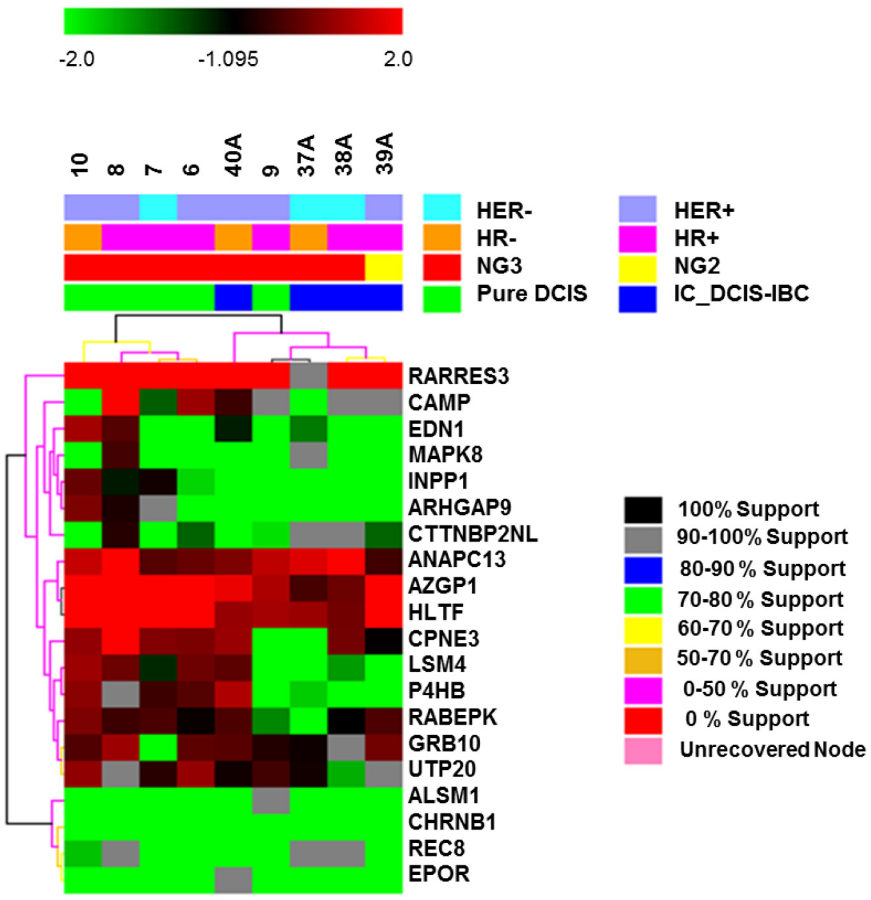

Supplementary Figure S4: Hierarchical clustering based on the expression profile of the 20 differentially expressed genes between pure DCIS and *in situ* component of DCIS-IBC samples of an independent group of samples. Unsupervised hierarchical clustering with Euclidean distance and average linkage of 9 tumor samples, including 5 pure DCIS (green) and 4 *in situ* components of DCIS-IBC matched-samples (blue). For each row (gene), the expression values were subtracted from the respective mean value of the row. The columns, rows, red, green and gray colors represent samples, genes, up and down regulated and not determined genes, respectively. The hierarchical clustering was based on log2-transformed expression values obtained from RT-qPCR validation. The expression profile was associated with the different invasion capabilities of pre-invasive cells and was able to discriminate 80% of the epithelial cell samples captured from pure DCIS from 100% of those captured from the *in situ* component of DCIS-IBC. *Abbreviations*: DCIS, ductal carcinoma *in situ*; IC_DCIS-IBC, *in situ* component of DCIS-IBC; HER2, human epidermal receptor 2; HR, hormonal receptor (estrogen and progesterone receptors); ND, FISH non-determined; NG, nuclear grade.

A.

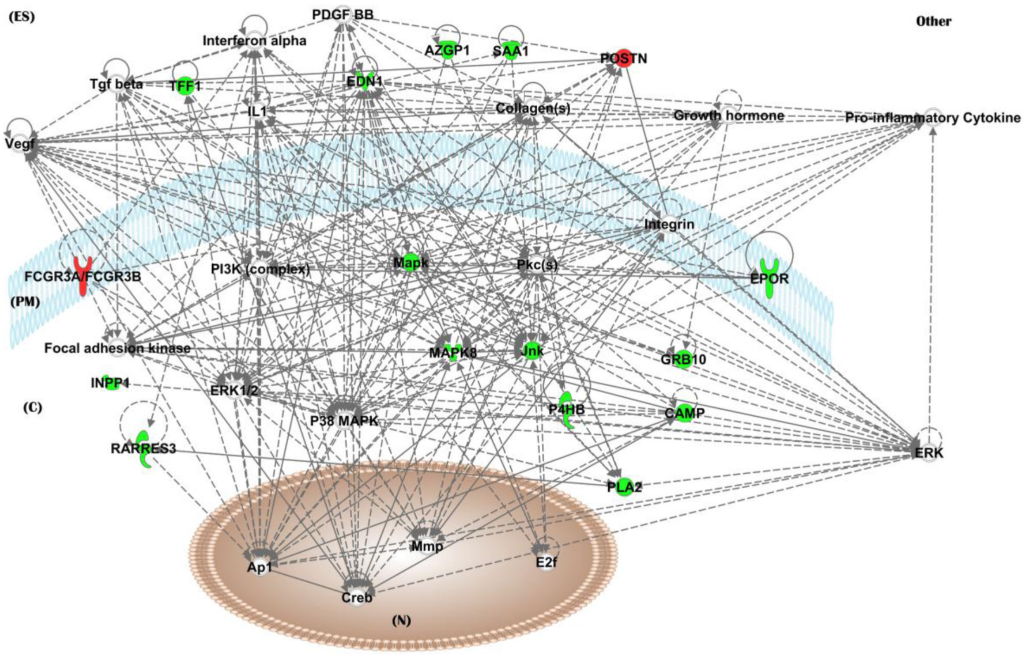

B.

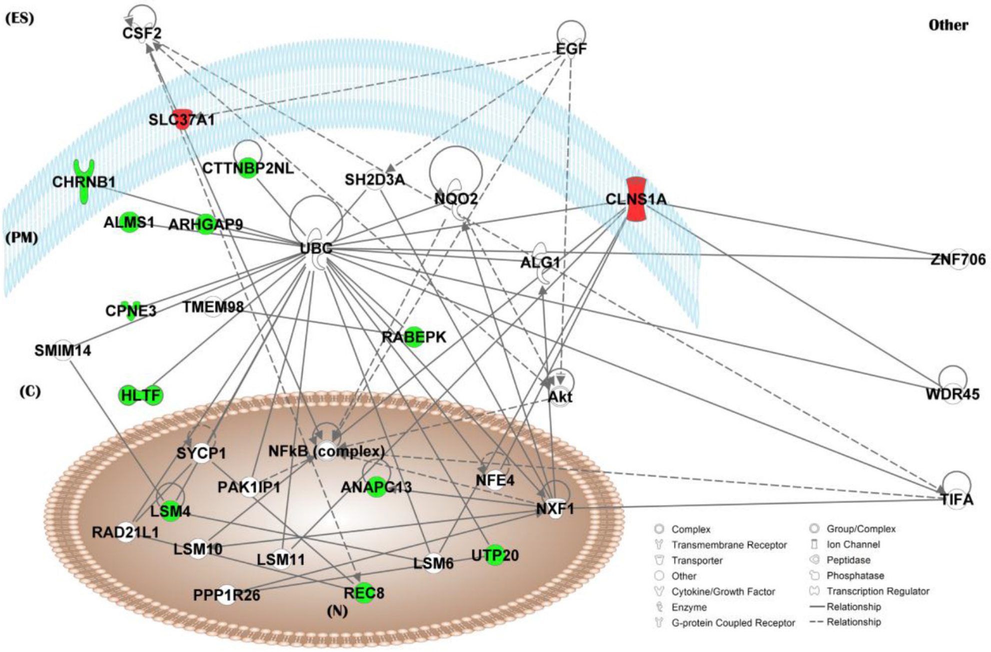

Supplementary Figure S5: Network and functional analysis by IPA of the 26 differentially expressed genes in the early and late stages of DCIS progression. A. Top scored gene network 1 (score 32) associated to cell death and survival, cell signaling, cellular function and maintenance. **B.** Top scored gene network 2 (score 32) associated to cell cycle, reproductive system development and function, cellular development. These networks are based on fold change values of the 26 differentially expressed genes validated by RT-qPCR. Gene network is represented as nodes and lines between two nodes. Continuous and dashed lines indicate direct and indirect interactions between molecules, respectively. Nodes labeled in red and green represent up- and down-regulation of gene expression, respectively. Empty nodes represent other genes automatically included by IPA due to their biological similarity. Nodes are displayed using various shapes that represent the functional class of the gene product. Abbreviations: (C), Cytoplasm; (ES), Extracellular Space; (N), Nucleus; (PM), Plasma Membrane.

REFERENCE

1. Castro NP, Osório C a BT, Torres C, Bastos EP, Mourão-Neto M, Soares F a, Brentani HP, Carraro DM. Evidence that molecular changes in cells occur before morphological alterations during the progression of breast ductal carcinoma. Breast Cancer Res [Internet]. 2008; 10: R87. doi: 10.1186/bcr2157.

Supplementary Table S1: Patient and tumor characteristics of clinical samples designated to each assay.

See Supplementary File 1

Supplementary Table S2: Differentially expressed genes between pure DCIS and *in situ* component of DCIS-IBC samples from cDNA microarray platforms selected for TLDA validation.

See Supplementary File 2

Supplementary Table S3: Differentially expressed genes in matched DCIS-IBC samples from cDNA microarray platforms selected for TLDA validation

Gene Symbol	FC Microarray	TLDA validation	FC TLDA
cDNA 2.3K platform			
CITED1	1.60		
MYC	-1.63	x	2.70
RIPK4	1.57	x	ND
SOX9	1.61	x	1.3E09
cDNA 4.8K platform			
ANKRD30A	1.52	x	1.4
BAMBI	-1.925		
C14orf109	-1.86		
C1orf24	1.6		
CCBL1	-1.95	x	-3.6
CNNM3	-1.64	x	-6.8
COL10A1	-1.96	x	6.00E+06
CRABP2	-1.54	x	1.2
CXCL13	1.53	x	2.6
DKFZp434L142	-1.51		
EFHD1	-1.51	x	-1.4
FBN1	-1.53		
FCGR3A[a]	-1.53	x	-3
GDF15	1.77	x	1.2
GIT1	-1.61	x	ND
GJA1	-1.81		
KIAA0368	-2.34	x	1.1
LATS2	-1.71		
LPL	1.51	x	1.1
LUM	-2.85	x	-1.2
MAGEH1	-1.74		
MRPS16	-2.05	x	1.8
POSTN[a]	-2.12	x	-8.5
PRLR	-2.07	x	1.1
RAB10	-1.62	x	-1.1
RDH-E2	2.21		
RG9MTD3	-1.63		

(*Continued*)

Gene Symbol	FC Microarray	TLDA validation	FC TLDA
SLC35A4	-1.5	x	-1
SLC37A1[a]	-2.26	x	-6.5
STK3	-2.06	x	1.5
TMEFF2	-1.68	x	ND
TSTA3	-1.59	x	-1.5
VMP1	-1.62	x	1.1

Differentially expressed genes from 4.8K and 2.3K cDNA microarray platforms (fold change ≥ |1.5| and P-value < 0.05). Twenty-seven genes were selected for RT-qPCR experiments (X). Positive and negative *fold change* values indicate increased and decreased expression in *in situ* component of DCIS-IBC, respectively.[a] Genes confirmed by TLDA assays (pairwise Student's t-test *fold change* ≥ |2| and *P*-value < 0.05). Abbreviations: DCIS, ductal carcinoma *in situ*; IBC, invasive breast carcinoma; FC, fold change; ND, non-detected amplification; TLDA, taqMan low density array.

Supplementary Table S4: List of the 171 genes with increased expression in DCIS subtracted cDNA library through RaSH approach.

See Supplementary File 3

Supplementary Table S5: List of the 214 genes with increased expression in IBC subtracted cDNA library through RaSH approach.

See Supplementary File 4
