# Supplementary material for: Epithelial cells captured from ductal carcinoma in situ reveal a gene expression signature associated with progression to invasive breast cancer
Source: Oncotarget. 2016 Sep 30;7(46):75672–84. doi: 10.18632/oncotarget.12352 (PMC5342769; doi:10.18632/oncotarget.12352)
Supplement: Supplementary file 2 [file oncotarget-07-75672-s002.docx]

**Table S1**

| **Patient and tumor characteristics of clinical samples designated to each assay** | | | | | | | |
| --- | --- | --- | --- | --- | --- | --- | --- |
| Specimen description | Age | pTNM | Nuclear grade | SBR grade | Molecular markers | Microarray platform/ RaSH | TLDA |
| Pure DCIS |  |  |  |  |  |  |  |
| 1 | 37 | TisN0M0 | 3 | ND | ER -/PR -/P53 +/ HER2 (0) | 2.3K^(a)^/4.8K^(a)^ | X ^(a)^ |
| 2 | 44 | TisN0M0 | 3 | ND | ER +/ PR +/ P53 -/ HER2 (0) | 2.3K^(a)^/4.8K ^(a)^ | X ^(a)^ |
| 3 | 43 | TisN0M0 | 3 | ND | ER +/ PR+/ P53-/ HER2 (3+) | 2.3K^(a)^/4.8K ^(a)^ | X ^(a)^ |
| 4 | 52 | TisN0M0 | 3 | ND | ER +/ PR+/ P53-/ HER2 (3+) | 4.8K ^(a)^ |  |
| 5 | 58 | TisN0M0 | 3 | ND | ER-/ PR-/P53-/HER2 (3+) | 2.3K^(a)^/4.8K ^(a)^ | X ^(a)^ |
| 6 | 65 | TisN0M0 | 3 | ND | ER+/PR+/HER2 (3+) |  | X ^(a)^ |
| 7 | 42 | TisN0M0 | 3 | ND | ER+/PR+/HER2 (0) |  | X ^(a)^ |
| 8 | 48 | TisN0M0 | 3 | ND | ER+/PR+/HER2 (3+) |  | X ^(a)^ |
| 9 | 58 | TisN0M0 | 3 | ND | ER+/PR+/HER2 (3+) |  | X ^(a)^ |
| 10 | 54 | TisN0M0 | 3 | ND | ER-/PR-/ HER2 (3+) |  | X ^(a)^ |
| *in situ* component of DCIS-IBC |  |  |  |  |  |  |  |
| 11A | 45 | T2N0M0 | 2 | II | ER -/ PR -/P53 -/ HER2 (3+) | 2.3K ^(b)^/4.8K ^(b)^ |  |
| 12A | 48 | T2N0M0 | 3 | ND | ER +/PR +/P53 +/HER2 (1+) | 2.3K ^(b)^/4.8K ^(a, b)^ | X ^(a, b)^ |
| 13A | 43 | T1cN0M0 | 3 | II | ER+/PR-/p53-/HER2(3+) | 2.3K ^(b)^/4.8K ^(b)^ | X ^(b)^ |
| 14A | 75 | T2N0M0 | 2 | II | ER+/PR+/p53+/HER2(2+) | 2.3K ^(b)^/4.8K ^(b)^ |  |
| 15A | 47 | T2N1M0 | 2 | I | ER+/PR+/p53-/HER2 (3+) | 4.8K ^(b)^ | X ^(b)^ |
| 16A | 54 | T2N0M0 | 3 | II | ER +/ PR +/ P53 +/ HER2 (3+) | 4.8K ^(b)^ |  |
| 17A | 34 | T1cN0M0 | 3 | ND | ER +/PR +/P53 +/HER2 (3+) | 2.3K ^(b)^/4.8K ^(a. b)^ | X ^(a, b)^ |
| 18A | 38 | T1cN0M0 | 3 | III | ER+/PR-/p53-/HER2 (-) | 4.8K ^(a, b)^ |  |
| 19A | 55 | T1N0M0 | 3 | ND | ER -/PR -/P53 +/HER2 (3+) | 2.3K ^(a)^ | X ^(a)^ |
| 20A | 71 | T4N2M0 | 3 | III | ER +/ PR +/ P53 +/ HER2 (2+) (-)* | 4.8K ^(b)^ |  |
| 21A | 43 | T2N0M0 | 3 | III | ER-/HER2 (+3) | 4.8K ^(a, b)^ |  |
| 22A | 54 | T3N1M0 | 3 | III | ER -/ PR - P 53 -/ HER2 (1+) |  | X ^(b)^ |
| 23A | 44 | T4bN1M0 | 2 | ND | ER +/PR +/P53 ND/ HER2 (1+) (-)* | 4.8K^(b)^ | X ^(a)^ |
| 24A | 57 | T2N2M0 | 2 | II | ER+/PR+/HER2 (2+) | 2.3K ^(a, b)^/4.8K ^(a, b)^ |  |
| 25A | 43 | T2N0M0 | 3 | ND | ER -/ PR -/ P53 +/ HER2 (3+) | 2.3K ^(a, b)^/4.8K ^(a, b)^ | X ^(a, b)^ |
| 26A | 44 | T1N1M0 | 3 | II | ER +/ PR + P53 -/ HER2 (1+) | 2.3K ^(b)^/4.8K ^(b)^ |  |
| 27A | 31 | T2N1M0 | 3 | II | ER -/ PR -/ P53 +/ HER2 (3+) | 2.3K ^(b)^/4.8K ^(b)^ | X ^(b)^ |
| 28A | 45 | T2N1M0 | 3 | II | ER-/PR-/P53+/HER (+3) | 2.3K ^(a)^ |  |
| 29A | 48 | T2N0M0 | 2 | ND | ER +/ PR +/ P53 -/ HER2 (2+) (-)* | 2.3K ^(a, b)^/4.8K ^(b)^ | X ^(a, b)^ |
| 30A | 73 | T3N0M0 | 2 | ND | ER +/ PR +/ P53 -/ HER2 (2+) (-)* | 2.3K ^(a)^/4.8K ^(a)^ | X ^(a)^ |
| 31A | 45 | T2N0M0 | 3 | ND | ER +/ PR + P53 +/ HER2 (2+) (-)* | 2.3K ^(a)^/4.8K ^(a)^ | X ^(a)^ |
| 32A | 48 | T2N1M0 | 3 | ND | ER -/ PR -/ P53 -/ HER2 (2+) (-)* | 2.3K ^(a)^/4.8K ^(a)^ | X ^(a)^ |
| 33A | 63 | T2N0M0 | 1 | ND | ER +/ PR -/ P53 -/ HER2 (2+) (ND)* | 2.3K ^(a)^ | X ^(a)^ |
| 34A | 39 | T1cN0M0 | 3 | ND | ER +/ PR+/ P53 ND/ HER2 (3+) | 2.3K ^(a)^ | X ^(a)^ |
| 35A | 49 | T1cN0M0 | 2 | ND | ER +/ PR +/ P53 ND/ HER2 (0) | 2.3K ^(a)^ | X ^(a)^ |
| 36A | 69 | T2N1M0 | 2 | ND | ER +/ PR +/ P53 ND/ HER2 (2+) (-)* | 2.3K ^(a)^ | X ^(a)^ |
| 37A | 54 | T4bN2M0 | 3 | ND | ER-/PR-/HER2 (1+) |  | X ^(a)^ |
| 38A | 67 | T2N2M0 | 3 | ND | ER+/PR+/HER2 (1+) |  | X ^(a, b)^ |
| 39A | 56 | T1N2M0 | 2 | ND | ER+/PR+/HER2 (3+) |  | X ^(a, b)^ |
| 40A | 56 | T2N2aM0 | 3 | ND | ER-/PR-/ HER2 (2+) (+)* |  | X ^(a)^ |
| 41A | 45 | pT4bN1M0 | 3 | II | ER+/PR+/p53+/HER2 (+1) | RaSH |  |
| 42A | 44 | pT1cN1M0 | 3 | II | ER+/PR+/p53-/HER2 (+1) | RaSH |  |
| IBC |  |  |  |  |  |  |  |
| 11B | 45 | T2N0M0 | 2 | II | ER -/ PR -/P53 -/ HER2 (3+) | 2.3K ^(b)^/4.8K ^(b)^ |  |
| 12B | 48 | T2N0M0 | 3 | ND | ER +/PR +/P53 +/HER2 (1+) | 2.3K ^(b)^/4.8K ^(b)^ | X ^(b)^ |
| 13B | 43 | T1cN0M0 | 3 | II | ER +/ PR -/ P53 -/ HER2 (3+) | 2.3K ^(b)^/4.8K ^(b)^ | X ^(b)^ |
| 14B | 75 | T2N0M0 | 2 | II | ER+/PR+/p53+/HER2 (2+) | 2.3K ^(b)^/4.8K ^(b)^ |  |
| 15B | 47 | T2N1M0 | 2 | I | ER+/PR+/p53-/HER2 (3+) | 4.8K ^(b)^ | X ^(b)^ |
| 16B | 54 | T2N0M0 | 3 | II | ER +/ PR +/ P53 +/ HER2 (3+) | 4.8K ^(b)^ |  |
| 17B | 34 | T1cN0M0 | 3 | ND | ER +/PR +/P53 +/HER2 (3+) | 2.3K ^(b)^/4.8K ^(b)^ | X ^(b)^ |
| 18B | 38 | T1cN0M0 | 3 | III | ER+/PR-/p53-/HER2 (-) | 4.8K ^(b)^ |  |
| 20B | 71 | T4N2M0 | 3 | III | ER +/ PR +/ P53 +/ HER2 (2+) (-)* | 4.8K ^(b)^ |  |
| 21B | 43 | T2N0M0 | 3 | III | ER-/HER2 (+3) | 4.8K ^(b)^ |  |
| 22B | 54 | T3N1M0 | 3 | III | ER -/ PR - P 53 -/ HER2 (1+) |  | X ^(b)^ |
| 23B | 44 | T4bN1M0 | 2 | ND | ER +/PR +/P53 ND/ HER2 (1+)(-)* | 4.8K ^(b)^ |  |
| 24B | 57 | T2N2M0 | 2 | II | ER+/PR+/HER2 (2+) | 2.3K ^(b)^/4.8K ^(b)^ |  |
| 25B | 43 | T2N0M0 | 2 | ND | ER -/ PR -/ P53 +/ HER2 (3+) | 2.3K ^(b)^/4.8K ^(b)^ | X ^(b)^ |
| 26B | 44 | T1N1M0 | 3 | II | ER +/ PR + P53 -/ HER2 (1+) | 2.3K ^(b)^/4.8K ^(b)^ |  |
| 27B | 31 | T2N1M0 | 3 | II | ER -/ PR -/ P53 +/ HER2 (3+) | 2.3K ^(b)^/4.8K ^(b)^ | X ^(b)^ |
| 29B | 48 | T2N0M0 | 2 | ND | ER +/ PR +/ P53 -/ HER2 (2+) (-)* | 2.3K ^(b)^/4.8K ^(b)^ | X ^(b)^ |
| 38B | 67 | T2N2M0 | 3 | ND | ER+/PR+/HER2 (1+) |  | X ^(b)^ |
| 39B | 56 | T1N2M0 | 2 | ND | ER+/PR+/HER2 (3+) |  | X ^(b)^ |
| 41B | 45 | pT4bN1M0 | 3 | II | ER+/PR+/p53+/HER2 (+1) | RaSH |  |
| 42B | 44 | pT1cN1M0 | 3 | II | ER+/PR+/p53-/HER2 (+1) | RaSH |  |

DCIS, ductal carcinoma *in situ*; DCIS-IBC, ductal carcinoma *in situ* that coexists with IBC (A); IBC, invasive breast carcinoma (B); pTNM, pathological TNM (tumor-node-metastasis) staging system; ER, estrogen receptor; PR, progesterone receptor; HER2, human epidermal growth factor receptor type 2; (-)*, FISH unamplified; (+)*, FISH amplified; (ND)*, FISH undetermined; SBR grade, Scarff-Bloom-Richardson grading system; ND, not determined; TLDA, TaqMan low density array; X, sample used in TLDA array; RaSH, rapid subtractive hybridization.

^(a)^ Comparison between pure DCIS and *in situ* component of DCIS/IBC. ^(b)^ Comparison between matched DCIS/IBC samples.
