# Supplementary material for: Epithelial cells captured from ductal carcinoma in situ reveal a gene expression signature associated with progression to invasive breast cancer
Source: Oncotarget. 2016 Sep 30;7(46):75672–84. doi: 10.18632/oncotarget.12352 (PMC5342769; doi:10.18632/oncotarget.12352)
Supplement: Supplementary file 3 [file oncotarget-07-75672-s003.docx]

**Table S2.**

| **Differentially expressed genes between pure DCIS and *in situ* component of DCIS-IBC samples from cDNA microarray platforms selected for TLDA validation** | | | | | | | |
| --- | --- | --- | --- | --- | --- | --- | --- |
| Gene Symbol | FC Microarray | TLDA validation | FC  TLDA | Gene Symbol | FC Microarray | TLDA validation | FC TLDA |
| *cDNA 2.3K platform* |  |  |  | *cDNA 4.8K platform* |  |  |  |
| *ADH4* | 3.20 | **x** | ND | *ADFP* | 2.79 | x | 1.22 |
| *ALDH1A2* | 2.77 |  |  | *ALMS1* *^a^* | 3.82 | x | 6.30 |
| *BRCA2* | 2.34 |  |  | *ANAPC13* *^a^* | 2.19 | x | 3.00 |
| *CALCA* | 2.84 |  |  | *ARHGAP19* *^b^* | 2.22 | x | 11.95 |
| *CAMK4* | 2.06 |  |  | *ARHGAP9* *^a^* | 2.92 | x | 10.20 |
| *CD79A* | 3.49 | **x** | 3.86 | *AZGP1* *^a^* | 2.94 | x | 4.30 |
| *CHRNA7* | 2.57 |  |  | *C16orf5* | 5.45 | x | 1.19 |
| *COL5A1* | 2.51 | **x** | -1.68 | *C3AR1* | 3.04 | x | -1.40 |
| *CR2* | 2.31 |  |  | *CAMP* *^a^* | 2.84 | x | 3.10 |
| *CSEN* | 3.07 |  |  | *CHRNB1* *^a^* | 5.11 | x | 3.10 |
| *CTSC* | 2.14 |  |  | *CHST10* | 5.07 | x | 1.30 |
| *CYP1A1* | 2.43 |  |  | *CLTCL1* *^b^* | 2.07 | x | 3.70 |
| *DLEC1* | 2.24 |  |  | *CORO1C* | -3.05 | x | 3.06 |
| *DLL1* | 2.60 | **x** | 6.58 | *CPNE3* *^a^* | 2.38 | x | 2.80 |
| *DSCR1L2* | 3.61 |  |  | *CTTNBP2NL* *^a^* | 4.84 | x | 4.80 |
| *EDN1* *^a^* | 2.48 | **x** | 5.76 | *DENND3* | 3.51 | x | 2.12 |
| *EIF4G2* | 2.36 |  |  | *DGCR2* | 2.96 | x | 1.41 |
| *FANCD2* | 5.50 | **x** | 8.85 | *DHX35* | 3.07 | x | 5,95 |
| *FCGR1A* | 3.31 |  |  | *EPOR* *^a^* | 2.99 | x | 10.51 |
| *FGF8* | 2.56 |  |  | *ERC1* | 3.86 | x | 1.37 |
| *GDAP1* | -3.60 | **x** | 1.05 | *FCN1* | 3.79 | x | 2.80 |
| *GRB10* *^a^* | 3.50 | **x** | 4.40 | *FGFBP1* | 3.28 | x | ND |
| *HOXA1* | 3.90 | **x** | ND | *FN3K* | 6.30 | x | 2.80 |
| *HOXA7* | 2.42 |  |  | *GBGT1* | 4.06 | x | 1.67 |
| *HOXB5* | 2.01 |  |  | *GOSR2* | 3.97 | x | 1.75 |
| *HTR2A* | 4.00 | **x** | 1.72 | *HLTF* *^a^* | 2.98 | x | 2,98 |
| *INPP1* *^a^* | 2.62 | **x** | 8.56 | *IFT57* | 3.30 | x | 2.00 |
| *ITPKC* | 4.95 | **x** | 3.91 | *IMMT* | 2.92 | x | 1.51 |
| *L1CAM* | 2.72 |  |  | *IRF8* | 4.33 | x | 2.62 |
| *LRAT* | 2.78 |  |  | *ITGB2* | 3.27 | x | 2.99 |
| *MAP2* | 2.30 |  |  | *KIAA0664* | 3.45 | x | 1.66 |
| *MAP3K12* | 2.58 |  |  | *LOX* | -2.01 | x | -4.21 |
| *MAPK8* *^a^* | 3.71 | **x** | 2.00 | *LPXN* | 3.20 | x | 2.47 |
| *MBP* | 2.90 | **x** | 1.58 | *LSM4* *^a^* | 2.85 | x | 3.53 |
| *MMP10* | -2.30 |  |  | *MBD3* | 4.58 | x | -2.76 |
| *MTA1* | 4.40 | **x** | 1.17 | *NEDD9* | 6.02 | x | 1.78 |
| *MYB* | -3.10 | **x** | -2.54 | *NGDN* | 2.59 | x | -1.02 |
| *NCAM2* | 3.00 | **x** | -186 | *NMU* | -4.12 | x | 1.38 |
| *NGFB* | 2.39 |  |  | *OSBPL7* | 4.91 | x | ND |
| *NPY* | 2.80 | **x** | ND | *P4HB* *^a^* | 3.01 | x | 21.313 |
| *NR0B2* | 3.34 |  |  | *PCDH10* | 3.00 | x | ND |
| *NRG2* | 3.30 | **x** | 98,24 | *PEPD* | 3.65 | x | 2.12 |
| *OPN1LW* | 5.20 | **x** | ND | *PIAS2* | 2.43 | x | 1.68 |
| *PAX4* | 2.70 | **x** | ND | *PMPCA* | 4.20 | x | 1.57 |
| *PAX6* | 5.50 | **x** | 8.98 | *PPTC7* | 2.92 | x | -1.08 |
| *PCDHA8* | 3.13 |  |  | *PTBP2* | -2.84 | x | 1.49 |
| *PRKACG* | 2.67 | **x** | 6.83 | *RABEPK* *^a^* | 4.94 | x | 2.17 |
| *PTCH* | 3.05 |  |  | *RAD51AP1* | -2.96 | x | 1.02 |
| *RBM6* | -2.61 |  |  | *RARRES3* *^a^* | 3.64 | x | 3.38 |
| *RCD-8* | -2.82 |  |  | *REC8* *^a^* | 3.37 | x | 9.50 |
| *RELN* | 3.50 | **x** | 6.97 | *SAMD4A* | 4.11 | x | 7.80 |
| *RORB* | 2.49 |  |  | *SLC6A20* | 3.54 | x | ND |
| *SALL1* | 2.32 |  |  | *SOX13* | 3.58 | x | 1.63 |
| *TNFRSF8* | 3.40 | **x** | ND | *STX11* | 3.18 | x | ND |
| *WISP2* | 3.30 |  | 4.28 | *SULF1* | -3.92 | x | -2.00 |
| *WNT1* | 2.60 | **x** | ND | *SYN1* | 2.97 | x | ND |
| *WNT10B* | 2.60 | **x** | ND | *TARDBP* | 4.03 | x | 6.02 |
|  |  |  |  | *TRAP1* | 3.50 | x | 1.48 |
|  |  |  |  | *TXNDC11* | 6.00 | x | 1.05 |
|  |  |  |  | *UTP20* *^a^* | 3.16 | x | 2.79 |
|  |  |  |  | *ZBTB5* | 2.82 | x | ND |

Differentially expressed genes from customized 2.3K and 4.8K [1] cDNA microarray platforms (fold change ≥ |2| and *P*-value < 0.05). Eighty-nine genes were selected for RT-qPCR experiments (X). Positive and negative fold change values indicate increased and decreased expression in pure DCIS, respectively. ^a^ Genes confirmed by TLDA assays (pairwise Student’s t-test fold change ≥ |2| and *P*-value < 0.05). ^b^ Genes confirmed by TLDA assays (pairwise Student’s t-Test fold change ≥ |2| and *P*-value < 0.05) but expressed < 50% of the samples, thus omitted. Abbreviations: DCIS, ductal carcinoma *in situ*; DCIS-IBC, *in situ* component of DCIS-IBC; IBC, invasive breast carcinoma; FC, fold change; ND, non-detected amplification; TLDA, taqMan low density array.
