# Supplementary material for: Epithelial cells captured from ductal carcinoma in situ reveal a gene expression signature associated with progression to invasive breast cancer
Source: Oncotarget. 2016 Sep 30;7(46):75672–84. doi: 10.18632/oncotarget.12352 (PMC5342769; doi:10.18632/oncotarget.12352)
Supplement: Supplementary file 4 [file oncotarget-07-75672-s004.docx]

**Table S4.**

| **List of the 171 genes with increased expression in DCIS subtracted cDNA library through RaSH approach.** | | | | | | | | |
| --- | --- | --- | --- | --- | --- | --- | --- | --- |
| Gene symbol | TLDA Validation | FC TLDA | Gene symbol | TLDA Validation | FC TLDA | Gene symbol | TLDA Validation | FC TLDA |
| *AA207202* |  |  | *CAT* | x | 1.25 | *MMADHC* | x | 1.03 |
| *AA358397* |  |  | *CB242937* |  |  | *NCOA7* |  |  |
| *ACTA2* |  |  | *CCDC22* |  |  | *NISCH* |  |  |
| *ACTB* |  |  | *CCND1* |  |  | *NPNT* | x | -14.45 |
| *AF448858* |  |  | *CD511652* |  |  | *NQO1* |  |  |
| *AI418471* |  |  | *CD687728* |  |  | *NR2C2* |  |  |
| *AI546885* |  |  | *CDC16* |  |  | *NUMBL* |  |  |
| *AI908823* |  |  | *CHI3L1* |  |  | *OGFRL1* |  |  |
| *AK023601* |  |  | *CK821278* |  |  | *OSBPL8* | x | 1.74 |
| *AL122062* |  |  | *CN414308* |  |  | *PCSK1* |  |  |
| *AL833615* |  |  | *CN414853* |  |  | *PER2* | x | -1.07 |
| *ALDH1A1* |  |  | *CN484301* |  |  | *PGM5P2* |  |  |
| *ALG13* |  |  | *CR736439* |  |  | *PICALM* |  |  |
| *ANKHD1* |  |  | *CV369880* |  |  | *PIGG* |  |  |
| *ANKRD30A* | x | 1.41 | *DA318042* |  |  | *PILRB* |  |  |
| *ANXA1* | x | 2.41 | *DA405060* |  |  | *PLA2G12A* | x | ND |
| *AOF2* |  |  | *DA489876* |  |  | *PLCB4* | x | 6.15 |
| *APLP2* | x | 1.64 | *DA645859* |  |  | *PLCG1* |  |  |
| *APOBEC3B* |  |  | *DA646975* |  |  | *PLP2* |  |  |
| *ARFGEF1* |  |  | *DA812605* |  |  | *PPIA* |  |  |
| *ASNSD1* |  |  | *DB324154* |  |  | *PPP1CA* |  |  |
| *ASXL1* |  |  | *DB347221* |  |  | *PPP4R1* |  |  |
| *AU148184* |  |  | *DCAKD* |  |  | *PRDX4* |  |  |
| *AV651843* |  |  | *DN913290* |  |  | *PROSER3* |  |  |
| *AW088654* |  |  | *DNAJA1* |  |  | *R22320* |  |  |
| *AW137203* |  |  | *DST* |  |  | *RAB6A* |  |  |
| *AW151986* |  |  | *E2F4* | x | -1.34 | *RGC32* |  |  |
| *AW293174* |  |  | *EIF3M* |  |  | *RNF103* | x | 1.27 |
| *AZGP1* |  |  | *EIF4G3* |  |  | *RTN4* |  |  |
| *BBS4* |  |  | *EPHX2* |  |  | *SAA1*^a^ | x | 5.13 |
| *BC033025* |  |  | *EPRS* |  |  | *SDHB* |  |  |
| *BE122677* |  |  | *FAF1* |  |  | *SEC63* |  |  |
| *BF678333* |  |  | *FAM210B* |  |  | *SEPHS2* |  |  |
| *BF751901* |  |  | *FAM38B* |  |  | *SFRP1* | x | 2.46 |
| *BF968136* |  |  | *FAM83D* |  |  | *SLC39A8* | x | 2.03 |
| *BG167469* |  |  | *FBXW4* | x | -3.46 | *SMOC2* |  |  |
| *BI224335* |  |  | *FEN1* |  |  | *SNAP91* |  |  |
| *BM932001* |  |  | *GNB2L1* |  |  | *SNTB2* |  |  |
| *BMPR2* |  |  | *GREB1* | x | ND | *SPEN* |  |  |
| *BP418539* |  |  | *GRN* |  |  | *SSR3* |  |  |
| *BQ435770* |  |  | *H13414* |  |  | *STATIP1* |  |  |
| *BRD7* |  |  | *H91436* |  |  | *STIP1* |  |  |
| *BRD8* |  |  | *HLA-B* | x | 1.32 | *SYF2* |  |  |
| *BRWD1* |  |  | *HLXB9* |  |  | *T09065* |  |  |
| *BTAF1* |  |  | *HNRPAB* |  |  | *TANK* |  |  |
| *BU676595* |  |  | *HSP90AA1* |  |  | *TATDN1* |  |  |
| *BU684023* |  |  | *IGF1* | x | -43.95 | *TCP1* |  |  |
| *BU839374* |  |  | *INADL* | x | -1.39 | *TFF1*^a^ | x | 4.06 |
| *BX089752* |  |  | *ITGB3BP* | x | 2.07 | *TRAPPC2* |  |  |
| *BX497700* |  |  | *KCTD3* |  |  | *TRIP12* |  |  |
| *BX538341* |  |  | *KLHDC2* |  |  | *UBAP2L* | x | -1.28 |
| *BX648473* |  |  | *KRT18* |  |  | *UFM1* |  |  |
| *BX649033* |  |  | *KRT19* |  |  | *UQCR* |  |  |
| *C1S* |  |  | *MALAT1* |  |  | *VEZT* |  |  |
| *CA415848* |  |  | *MATN3* |  |  | *WDR33* | x | 1.02 |
| *CAPN2* |  |  | *METTL3* |  |  | *WDR42A* |  |  |
| *CASC4* |  |  | *MIR205HG* |  |  | *XRN2* |  |  |

Positive and negative fold change values indicate increased and decreased expression in *in situ* and invasive components of matched DCIS-IBC samples, respectively. Twenty-four genes were randomly selected RT-qPCR experiments (X). ^a^ Genes confirmed by TLDA assay (pairwise Student’s t-test fold change ≥ 2 and *P*-value < 0.05). Abbreviations: DCIS, ductal carcinoma *in situ*; DCIS-IBC, *in situ* component of DCIS-IBC; FC, fold change; IBC, invasive breast carcinoma; ND, non-detected amplification; NA, not applicable; RaSH, rapid subtractive hybridization; TLDA, taqMan low density array.
