# Supplementary material for: Epithelial cells captured from ductal carcinoma in situ reveal a gene expression signature associated with progression to invasive breast cancer
Source: Oncotarget. 2016 Sep 30;7(46):75672–84. doi: 10.18632/oncotarget.12352 (PMC5342769; doi:10.18632/oncotarget.12352)
Supplement: Supplementary file 5 [file oncotarget-07-75672-s005.docx]

**Table S5.**

| **List of the 214 genes with increased expression in IBC subtracted cDNA library through RaSH approach.** | | | | | | | | |
| --- | --- | --- | --- | --- | --- | --- | --- | --- |
| Gene symbol | TLDA Validation | FC TLDA | Gene symbol | TLDA Validation | FC TLDA | Gene symbol | TLDA Validation | FC TLDA |
| *AB046792* |  |  | *DN915024* |  |  | *PITPNA* |  |  |
| *AB209172* |  |  | *EEF1A1* |  |  | *POLS* |  |  |
| *ACAD11* |  |  | *EGR2* |  |  | *POMP* |  |  |
| *ACLY* |  |  | *EIF1* |  |  | *POSTN* |  |  |
| *ACOT2* |  |  | *EIF3S5* |  |  | *PPGB* |  |  |
| *AI569340* |  |  | *EIF4A2* | x | 2.11 | *PPIC* |  |  |
| *AI570286* |  |  | *EIF4G2* |  |  | *PPP1CB* |  |  |
| *AI925925* |  |  | *EMC1* |  |  | *PRDM4* |  |  |
| *AK023481* |  |  | *ENTPD5* |  |  | *PRKCBP1* |  |  |
| *AK026101* |  |  | *EXT2* |  |  | *PRKD2* |  |  |
| *AK074908* |  |  | *FAHD1* |  |  | *PRPF18* |  |  |
| *AL833401* |  |  | *FASN* |  |  | *PSMD12* |  |  |
| *APC* |  |  | *FCER1G* |  |  | *PTTG1IP* |  |  |
| *ARHGEF16* |  |  | *FNIP1* |  |  | *PYGL* |  |  |
| *ARID4B* | x | -1.4 | *FST* |  |  | *RAB13* |  |  |
| *ARS2* |  |  | *FTH1* | x | 1.64 | *RAB22A* | x | 1.98 |
| *ATF4* |  |  | *FUS* |  |  | *RAI17* |  |  |
| *ATG13* |  |  | *FUT8* |  |  | *RAP1A* |  |  |
| *ATP5E* |  |  | *G1P3* |  |  | *RBBP8* |  |  |
| *ATXN10* |  |  | *GEMIN6* |  |  | *RDBP* | x | 1.16 |
| *AU136967* |  |  | *GLUL* |  |  | *SAR1A* |  |  |
| *AV719766* |  |  | *GNAS* |  |  | *SAT* |  |  |
| *AW835509* |  |  | *GNB2* | x | 2.76 | *SERPINA3* |  |  |
| *AW956036* |  |  | *GPRC5C* |  |  | *SF3B14* |  |  |
| *B3GNT6* |  |  | *GRSF1* |  |  | *SFRS11* |  |  |
| *BAG3* |  |  | *GSTM3* | x | ND | *SH3BGRL* |  |  |
| *BC053563* |  |  | *GTF2F2* |  |  | *SHTN1* |  |  |
| *BF947412* |  |  | *HIP1* |  |  | *SKP1A* |  |  |
| *BG399854* |  |  | *HLA-DOA* |  |  | *SLC12A2* |  |  |
| *BG681172* |  |  | *HLA-DRA* |  |  | *SLC1A4* |  |  |
| *BM971791* |  |  | *HSP70B* |  |  | *SLC25A44* |  |  |
| *BM990717* |  |  | *HSP90AB1* |  |  | *SLC40A1* |  |  |
| *BQ303893* |  |  | *HSP90B1* |  |  | *SLC44A1* | x | 2.98 |
| *BQ690425* |  |  | *IGFBP7* |  |  | *SMARCE1* |  |  |
| *BTF3* |  |  | *IRXL1* |  |  | *SMIM14* |  |  |
| *BTN3A3* |  |  | *ITPR1* | x | -2.18 | *SNF1LK* |  |  |
| *BX098142* |  |  | *IWS1* |  |  | *SP1* |  |  |
| *BX103072* |  |  | *JTB* |  |  | *SPAG6* |  |  |
| *BX109074* |  |  | *KBTBD2* |  |  | *SPCS2* |  |  |
| *C1orf27* | x | -1.38 | *KIAA1324* |  |  | *SRP9* |  |  |
| *C3orf62* |  |  | *LASS6* |  |  | *SRPK1* |  |  |
| *C8orf76* |  |  | *MAL2* |  |  | *ST6GAL1* |  |  |
| *CA12* | x | -1.57 | *MAP3K1* |  |  | *STEAP2* |  |  |
| *CA775602* |  |  | *MBNL1* |  |  | *STOM* |  |  |
| *CANX* |  |  | *MEMO1* |  |  | *TAPBP* |  |  |
| *CCT8* |  |  | *MLEC* |  |  | *THBS2* |  |  |
| *CD46* |  |  | *MLL5* |  |  | *TLOC1* |  |  |
| *CD518345* |  |  | *MLPH* |  |  | *TMED10* |  |  |
| *CD59* |  |  | *MORF4L1* |  |  | *TMEM106B* |  |  |
| *CD656502* |  |  | *MPHOSPH8* |  |  | *TMEM98* |  |  |
| *CD74* |  |  | *MTL5* |  |  | *TMTC3* |  |  |
| *CDK5RAP3* |  |  | *MTMR11* |  |  | *TNFAIP3* |  |  |
| *CFB* |  |  | *MYDGF* |  |  | *TPM1* |  |  |
| *CHMP4A* |  |  | *MYEOV2* |  |  | *TRIP6* | x | -1.08 |
| *CHST15* | x | 1.98 | *NAP1L1* | x | 1.38 | *TSN* |  |  |
| *CILP* |  |  | *NCOR1* |  |  | *UBA1* |  |  |
| *CLNS1A*^a^ | x | -2.77 | *NDUFB2* |  |  | *USP4* |  |  |
| *CN293284* |  |  | *NFAT5* |  |  | *USP9X* |  |  |
| *CNOT6* |  |  | *NKTR* |  |  | *VEGF* |  |  |
| *COPB2* |  |  | *NMI* |  |  | *VPS13A* |  |  |
| *COPS6* |  |  | *NOL10* |  |  | *VPS24* |  |  |
| *COX4I1* |  |  | *ORC5L* |  |  | *WDR61* |  |  |
| *CSNK1E* |  |  | *PAIP2* |  |  | *WDR76* |  |  |
| *CTGF* | x | -4.12 | *PARP6* |  |  | *YARS* |  |  |
| *CTSH* |  |  | *PCBP1* |  |  | *YWHAZ* |  |  |
| *CYFIP1* |  |  | *PCBP2* |  |  | *ZBTB8A* |  |  |
| *DA077127* |  |  | *PCMTD1* |  |  | *ZNF148* |  |  |
| *DA393184* |  |  | *PEBP1* |  |  | *ZNF160* |  |  |
| *DDX21* |  |  | *PEG3* |  |  | *ZNF281* |  |  |
| *DDX58* | x | -2.63 | *PEX7* |  |  | *ZNF317* |  |  |
| *DIP2C* |  |  | *PHB2* |  |  |  |  |  |
| *DIS3L* | x | -3.8 | *PIGY* |  |  |  |  |  |

Positive and negative fold change values indicate genes with increased expression in *in situ* and invasive components of matched DCIS-IBC samples, respectively. Eighteen genes were randomly selected for RT-qPCR experiments (X). ^a^ Genes confirmed by TLDA assay (pairwise Student’s t-test fold change ≥ |2| and *P*-value < 0.05). Abbreviations: DCIS, ductal carcinoma *in situ*; DCIS-IBC, *in situ* component of DCIS-IBC; IBC, invasive breast carcinoma; FC, fold change; ND, non-detected amplification; RaSH, rapid subtractive hybridization; TLDA, taqMan low density Array.
